# The interplay between executive functions and updating predictive representations

**DOI:** 10.1038/s41598-025-14876-2

**Published:** 2025-08-20

**Authors:** Felipe Pedraza, Teodóra Vékony, Bence C. Farkas, Frederic Haesebaert, Romane Phelipon, Imola Mihalecz, Karolina Janacsek, Barbara Tillmann, Royce Anders, Gaën Plancher, Dezso Nemeth

**Affiliations:** 1 Laboratoire d’Étude des Mécanismes Cognitifs, Université Lumière Lyon 2, Bron, France; 2 Centre de Recherche en Neurosciences de Lyon, INSERM, CNRS, Université Claude Bernard Lyon 1, CRNL U1028 UMR5292, Bron, France; 3 Gran Canaria Cognitive Research Center, Department of Education and Psychology, Atlántico Medio University, Las Palmas de Gran Canaria, Spain; 4 UVSQ, INSERM, CESP, Université Paris-Saclay, Villejuif, France; 5 Pôle Pilotage activités et Projets, Direction Générale Adjointe Enfance Famille Santé (DGAEFS), Guyancourt, France; 6 Centre de Recherche en Épidémiologie et en Santé des Populations, INSERM U1018, Université Paris-Saclay, Université Versailles Saint-Quentin, Paris, France; 7 Centre for Thinking and Learning, Institute for Lifecourse Development, School of Human Sciences, Faculty of Education, Health and Human Sciences, University of Greenwich, London, UK; 8 Institute of Psychology, ELTE Eötvös Loránd University, Budapest, Hungary; 9 Université Bourgogne Europe, CNRS, LEAD UMR5022, Dijon, France; 10 EPSYLON Laboratory, Department of Psychology, University Paul Valéry, Montpellier, France; 11 Institut Universitaire de France (IUF), Paris, France; 12 BML-NAP Research Group, Institute of Psychology, Eötvös Loránd University & Institute of Cognitive Neuroscience and Psychology, HUN-REN Research Centre for Natural Sciences, Budapest, Hungary

**Keywords:** Human behaviour, Neuroscience

## Abstract

Modifying habits, particularly unwanted behaviors, is often challenging. Cognitive research has focused on understanding the mechanisms underlying habit formation and how habits can be rewired. A key mechanism is statistical learning, the continuous, implicit extraction of probabilistic patterns from the environment, which forms the basis of predictive processing. However, the interplay between executive functions (EF) and the rewiring – or updating – of these probabilistic representations remains largely unexplored. To address this gap, we conducted an experiment consisting of four sessions: (1) Learning Phase – acquisition of probabilistic representations, (2) Rewiring Phase – updating these probabilistic representations, (3) Retrieval Phase – accessing learned representations, and (4) EF assessment, targeting five key aspects: attentional control, inhibition, working memory, flexibility, and verbal fluency. We focused on the relationship between these EF measures and the updating of previously acquired knowledge using an interindividual differences approach. Our results revealed a positive relationship between rewiring and inhibition, suggesting that better inhibitory control may facilitate the adaptive restructuring of probabilistic predictive representations. Conversely, a negative relationship was identified between rewiring and semantic fluency, implying that certain underlying aspects of verbal fluency tasks, such as access to long-term memory representations, may hinder the updating process. We interpret this relationship through the lens of competitive memory network models. Our findings indicate that the rewiring of implicit probabilistic representations is a multifaceted cognitive process requiring both the suppression of proactive interference from prior knowledge through cognitive inhibition and a strong reliance on model-free functioning.

**Supplementary Information:**

The online version contains supplementary material available at 10.1038/s41598-025-14876-2.

## Introduction

Contemporary challenges like the pandemic, climate change, and rapid societal shifts result in significant environmental changes, often fostering problematic habits and maladaptive behaviors^1^. In this context, the detailed comprehension of the neurocognitive dynamics underlying habit formation and the way we modify them has become a research question of the utmost importance. Traditionally, habits have been conceptualized as automatic links between stimuli and responses^2^ indifferent to the outcome value of the response, which distinguishes them from goal-directed behaviors in non-human animal studies^3^. In the context of humans, habits take on a more intricate nature, defined by a set of behavioral attributes^4–6^. They are acquired gradually through associative learning processes over extended periods of practice, frequently without conscious awareness^7^. Once established, these habits can be executed with minimal thought or attention, essentially operating automatically^8^. Numerous human studies have focused on the environmental contingencies fostering successful habit updating or rewiring^9–12^. These studies commonly emphasize the modification of context cues as a primary strategy for rewiring habitual responses. Building upon the premise that habit change generally involves the strategic adjustment of environmental contingencies, we turn our attention to the neuropsychological processes that shape habit formation and modification. In this article, we focus on uncovering the internal cognitive mechanisms that govern habit formation and modification. To achieve this, we examine how interindividual differences in cognitive profiles influence habit change through the lens of a probabilistic learning task.

Statistical learning (SL) stands out as a fundamental element of the intricate process of habit formation^13–15^. SL refers to the cognitive function that allows the implicit extraction of probabilistic regularities from the environment, even in the absence of explicit intention, feedback, or rewards^16–18^. This cognitive mechanism plays a pivotal role in predictive processing and contributes significantly to the acquisition of diverse skills, including language^19–21^motor^22,23^, musical^24–26^, and social abilities^27–29^. In the context of habit formation, SL fortifies the probabilistic connections between environmental cues and the invoked associated responses that constitute habitual behaviors^30,31^, and facilitates the development of predictions of upcoming events, leading to shorter reaction times for expected outcomes^26^.

For instance, consider the habitual actions of a French driver. Through SL, this driver unconsciously forms associations between the likely locations of relevant stimuli at intersections and the corresponding direction to turn their gaze. Consequently, it becomes second nature for them to instinctively look left at a crossing. Conversely, a British driver, also influenced by learned probabilistic associations, automatically looks right at a crossing^32^. A potential challenge arises when a French driver, accustomed to the habitual association of relevant stimuli emerging from the left at intersections, travels to England, where relevant stimuli are more likely to appear from the right. To adapt effectively, the French driver must update or rewire the previously acquired probabilistic representations, which might prove difficult^33^. Elucidating the neurocognitive processes involved in adapting to new or altered environments is crucial to understanding how probabilistic representations are updated.

Updating implicit probabilistic representations poses several challenges. Firstly, because these representations are largely unconscious, individuals may lack explicit awareness of what needs to be modified^34^, which can hinder rewiring. Secondly, SL has been characterized as a resilient cognitive mechanism, leading to representations that are resistant to forgetting^35^. While this robustness is beneficial for maintaining learned associations, it can be a barrier to modifying existing representations. Thirdly, this robustness may cause proactive interference^36^, where existing strong associations interfere with the encoding of novel probabilistic relationships, creating a cognitive obstacle to effectively adapt to environmental changes.

Previous research on rewiring implicit probabilistic information investigated whether providing explicit instructions could enhance the effectiveness of the rewiring process^14^. While the results indicated that explicit instructions did improve rewiring, they also uncovered that the updating process could occur implicitly as well. Participants were able to adapt and rewire their implicit probabilistic representations without explicit guidance, underscoring the flexibility and adaptability of the underlying cognitive processes.

The examination of rewiring implicit probabilistic representations has also been approached through the lens of dual-process neuro-computational models of learning^37^. These models differentiate between model-free and model-based algorithms, with the former relying solely on recent outcomes and the latter building and utilizing a model of the environment to guide choices more flexibly, but at a higher computational cost^38,39^. The brain is postulated to alternate between these two learning systems, both within the completion of individual tasks^40–42^ and over the lifespan^43–45^. In their investigation, Kurdi and colleagues employed a stimulus reevaluation protocol based on the Implicit Association Test^45^ to examine the potential for updating implicit and explicit representations via model-based and model-free learning mechanisms. Their findings showed that while the updating of explicit probabilistic representations was influenced by both model-based and model-free processes, implicit probabilistic representations were updated exclusively through model-free processes.

Cognitive control functions, also known as executive functions (EF), may be a crucial tool for effectively rewiring probabilistic representations. EF is particularly important in non-habitual situations and includes cognitive functions such as attentional control, cognitive flexibility, cognitive inhibition, and working memory updating^46–48^. These functions are indispensable for model-based processes^49^ and for the flexible regulation of behavior^46,50^, making them pivotal in habit change^51,52^. For instance, a study demonstrated that cognitive remediation therapy aimed at enhancing EF could effectively disrupt bad habits associated with obesity^52^. Investigations into the neural underpinnings of EF consistently underscore the significant involvement of the prefrontal cortex (PFC)^53–57^. This reliance on the PFC is further evident at an interindividual level, where superior EF is associated with greater prefrontal activity^58^, and increased functional connectivity between the PFC and other brain regions^59^. Our focus here centers on examining whether and how interindividual differences in EF may influence the rewiring of implicit probabilistic representations.

While the association between EF and habit change may suggest a positive correlation, the relationship between EF and the cognitive processes underlying habit formation is more nuanced. Greater PFC activity has been consistently shown to hinder the acquisition of implicit probabilistic information. Disruption of PFC activity through interventions such as transcranial magnetic stimulation^60^, hypnosis^61^, cognitive fatigue induction^62^, or dual-tasking^63,64^ has resulted in improved acquisition of probabilistic representations. Neuroimaging studies have revealed that decreased PFC activity^65^ and decreased PFC functional connectivity^66^is also associated with enhanced SL. Similarly, at a cognitive level, studies have shown a negative relationship between EF and SL. For example, one study demonstrated that alcohol-dependent patients with weaker EF exhibited enhanced SL capacity^67^. Similar results were a study that offered an internal replication with healthy participants^68^. These studies suggest that while initial SL may rely on model-free learning algorithms, EF may facilitate model-based learning algorithms and align with the competition hypothesis between model-based and model-free learning. According to this hypothesis, these systems alternate in the brain during task completion, with greater reliance on one reducing reliance on the other^39,42,69^. The PFC plays a crucial role in mediating between model-based and model-free systems, with the latter serving as the default but able to be overridden by cognitive control mechanisms implemented by the PFC^41,70^. However, it is important to note that these studies mainly focus on the relationship between cognitive control and SL during initial knowledge acquisition. The relationship between cognitive control and the updating of probabilistic knowledge remains unclear.

To our knowledge, only one study has investigated the influence of EF on the rewiring of probabilistic representations to date^15^. This study specifically examined the impact of cognitive control, as manifested by active inhibition, on rewiring. The findings revealed that participants, while actively attempting to inhibit unwanted behaviors, inadvertently impeded their ability to acquire new probabilistic knowledge. Simultaneously, they reinforced previously acquired knowledge, suggesting that cognitive control may hinder the rewiring process. However, this study did not examine how individual differences in EF capacities affect rewiring. Moreover, cognitive control functions extend beyond inhibitory control. Therefore, there remains a critical need for further investigation into how various EF interact with the rewiring of implicit probabilistic representations. Understanding these interactions is crucial for gaining insight into the neuropsychological dynamics underlying habit change.

In this study, our objective was to investigate the relationship between EF and the updating of implicit probabilistic knowledge. We aimed to characterize these cognitive processes at the interindividual level using a four-session experimental protocol comprised of empirically validated tasks and measures of EF and SL (see Methods section). In the first session, participants underwent a learning phase in which they acquired implicit probabilistic representations in a visuo-motor probabilistic sequence learning task known as the Alternating Serial Reaction Time (ASRT) task. In the second session, participants completed a rewiring phase, where structural changes to the ASRT task sequence required them to update or rewire their initially acquired probabilistic representations. On the third day, during the retrieval phase, participants were tested on both their initial and rewired knowledge. The fourth session was dedicated to assessing participants’ EF capacities using a battery of validated and reliable neuropsychological tasks targeting various aspects of cognitive control. Drawing upon the framework of competitive neurocognitive systems and existing literature, we hypothesized a negative correlation between EF capacities and the rewiring of implicit probabilistic representations.

## Methods

### Participants

Seventy-nine healthy young adults were recruited through online advertisement with eligibility based on the following criteria: participants had to be right-handed, aged under 35 years, and have no or limited musical training (less than 10 years of practice). Participants declared having no active neurological or psychiatric conditions, and not taking any psychoactive medication. Of the 79 individuals initially recruited, three did not complete the four-session experiment and were excluded from the analysis. To accurately assess the relationship between EF and rewiring performance, participants had to demonstrate acquisition of implicit probabilistic representations that could be rewired (see *Statistical analysis section for the selection criteria*). Of the 76 remaining participants, 17 did not exhibit SL during the Learning Phase. This proportion of non-learners is consistent with previous studies examining inter-individual differences in the ASRT task^67,68,71,72^, addressing concerns about task sensitivity in inter-individual SL studies^73^. Thus, the data from the remaining 59 participants (32 females, M _age_ = 22.51 years; SD _age_ = 2.92 years; M _education_ = 15.34 years; SD _education_ = 1.64 years) were included in the analyses. Exploratory analyses confirmed that within our sample of young adults, there were no significant correlations between age and our primary learning or EF measures, nor were there significant differences between genders. Thus, age and gender were not included as covariates in subsequent analyses. Further details on participant characteristics can be found in Supplementary Table 1. The relevant institutional review board (i.e., the “Comité de Protection des Personnes, CPP Est I” ID: RCB 2019-A02510-57) gave ethical approval for the study. The authors confirm that all research was performed in accordance with relevant guidelines and in accordance with the Declaration of Helsinki. All participants provided signed informed consent agreements and received a 200 euros financial compensation for their participation in the four sessions of the experiment.

### Tasks

#### Measure of SL and rewiring: the alternating serial reaction time (ASRT) task

**Fig. 1:**
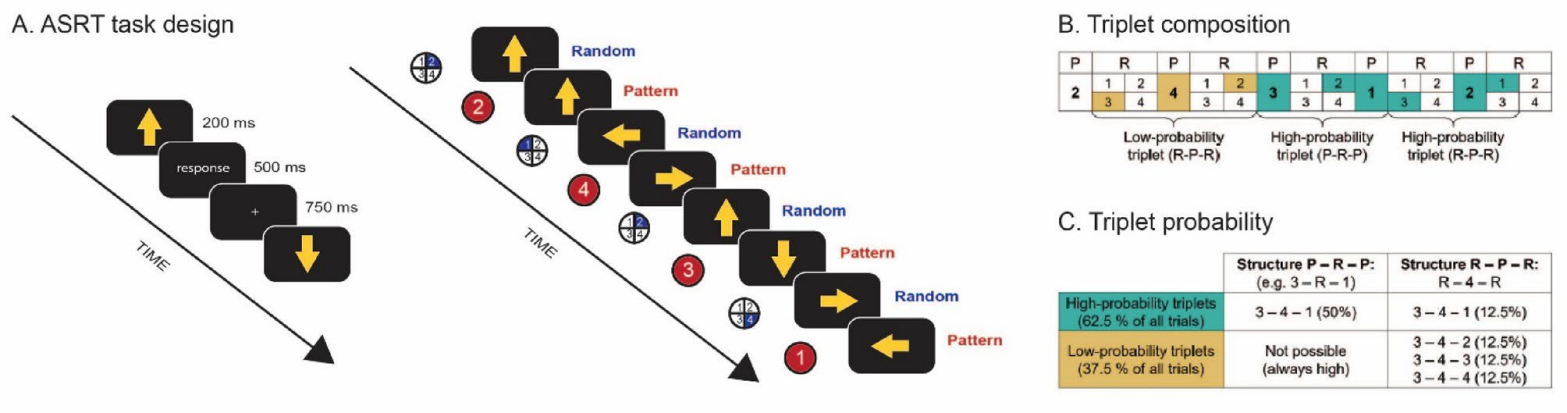
ASRT task structure. (**A**) ASRT task design. The presentation stimuli (yellow arrows, indicating one of the four cardinal directions), followed an eight-element sequence alternating between pattern (P) and random (R) stimuli. This sequence was presented 10 times per block. Here we illustrate an example of a 2-R-4-R-3-R-1-R sequence. (**B**) Triplet composition. Each stimuli position can be coded with a number. Here we have 1 = left, 2 = up, 3 = down, 4 = right. From the alternating structure of the sequence between P and R stimuli, certain triplets (sequences of three consecutive stimuli) have a higher probability of occurrence than others. For example, in the 2-R-4-R-3-R-1-R sequence, the triplet 3-2-1 has a higher probability of occurrence since it can be formed in P-R-P and R-P-R structures. The triplet 3-4-2 has a lower probability of occurrence since it can only appear in R-P-R structures. High-probability triplets could end in either a pattern or random element, while low-probability triplets always concluded with a random element. High- and low-probability triplets are denoted in green and yellow, respectively. (**C**) Triplet probability. High-probability triplets account for 62.5% of all trials and low-probability triplets account for the rest. Notably, for each high-probability triplet (e.g., 3-4-1), there are low-probability triplets with three different last elements (e.g., 3-4-2, 3-4-3 and 3-4-4). This structure overall results in each high-probability triplet being five times more likely to occur than the low-probability triplets.

To evaluate the acquisition and rewiring of implicit probabilistic information, we utilized a visuomotor SL task that has been empirically validated^71^and previously established as reliable^72^, known as the Alternating Serial Reaction Time (ASRT) task^74^. Employing a modified version of the ASRT task (Fig. 1A), our study adhered to a structured three-session paradigm^14^, designed to investigate the phases of acquisition, rewiring, and consolidation of implicit probabilistic information.

##### Session 1: learning phase

During the Learning Phase, participants performed Sequence A of the ASRT task. In each trial, a yellow arrow appeared at the center of the screen for 200 ms, pointing in one of four possible directions (left, up, down, or right). This was followed by a fixation cross displayed for 500 ms. Participants were instructed to respond as quickly as possible by pressing the button corresponding to the arrow’s direction on a Cedrus RB-530 response box. Finger placement was controlled as follows: the left index finger for the up button, the right thumb for the down button, the right index finger for the right button, and the left thumb for the left button. Correct responses were followed by another 750 ms display of the fixation cross, while no response or incorrect responses were met with a 500 ms display of an exclamation mark or an “X,” respectively, followed by a 250 ms fixation cross. Each block consisted of 85 stimuli, with the initial five presented randomly for practice, followed by 10 repetitions of the eight-element alternating sequence. The Learning Phase comprised 25 blocks of the ASRT task.

Unbeknownst to participants, the stimuli followed a structured sequence alternating between predefined pattern elements (P) and random elements (R) (e.g., 2-R-4-R-3-R-1-R, where the numbers represent the direction of the yellow arrows: 1 for left, 2 for up, 3 for down, 4 for right, and “R” indicating a randomly chosen direction out of the four possibilities). This structure resulted in certain triplets (runs of three trials) having a higher probability of occurrence than others (Fig. 1B). For instance, in the 2-R-4-R-3-R-1-R sequence, triplets like (2-3-4) or (3-4-1) were more likely to occur than triplets like (2-3-2) or (4-3-1) since the former triplets could appear in both P-R-P or in R-P-R structures whereas the latter triplets could only appear in R-P-R structures Ü(Fig. 1C). Consequently, the former triplets were five times more likely to occur than the latter, hence they were categorized as high-probability (H) and low-probability (L) triplets, respectively. Previous studies using the ASRT task consistently demonstrate that, with practice, participants respond more quickly to the last element of high-probability triplets compared to low-probability triplets, indicating SL^35,44,75^. Importantly, participants typically do not develop explicit knowledge of the sequence structure despite this learning effect^75–77^. Thus, learning in the task is assessed by calculating the reaction time difference between the last elements of high-probability and low-probability triplets, and this learning measure reflects implicit SL.

##### Session 2: rewiring phase

**Fig. 2:**
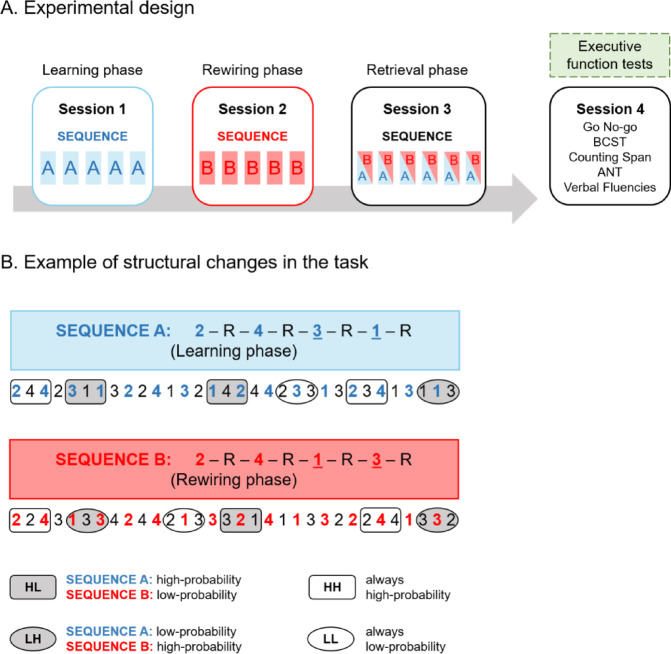
Experimental design and sequence changes. (**A**) Experimental design. The experiment comprised four sessions: the first three were held 24 h apart, and the fourth was independent. Sessions one to three included Learning, Rewiring, and Retrieval phases. In the Learning Phase, participants completed 25 blocks of Sequence A of the Alternating Serial Reaction Time (ASRT) task to acquire initial probabilistic representations. During the Rewiring Phase, structural changes introduced Sequence B, and participants updated their representations over 25 ASRT blocks. The Retrieval Phase involved alternating between Sequences A and B every five blocks for 30 blocks, probing knowledge of both sequences. The starting order of the sequences in the Retrieval Phase was counterbalanced across participants (half started with Sequence A and the other half started with Sequence B). In the figure, blue, red, and half-colored rectangles represent a bin (five blocks) of the ASRT task. The fourth session included EF assessments with five tasks: Go/No-Go, Berg Card Sorting Test (BCST), Counting Span, Attention Network Test (ANT), and three Verbal Fluency tasks (action, semantic, and lexical fluency). (**B**) Example of structural changes in the ASRT task. The figure shows the stimulus order for Sequences A and B, coded as numbers, with predetermined stimuli in color alternating with random stimuli (R in black). The alternating structure resulted in high- and low-probability triplets. The Rewiring Phase modified Sequence A into Sequence B, altering triplet probabilities. Specifically, 75% of initially high-probability triplets became low-probability (HL trials, gray squares), replaced by new high-probability triplets that were initially low-probability in Sequence A (LH trials, gray ovals). Other triplets maintained their original probability, being either low-probability (LL trials, white ovals) or high-probability (HH trials, white squares) in both phases.

During the Rewiring Phase, participants completed 25 blocks of Sequence B of the ASRT task, which involved structural modifications to the original sequence of the Learning Phase (Fig. 2A). Specifically, the last two pattern elements (P) of the original eight-element sequence were inverted. For example, if Sequence A was structured as 2-R-4-R-3-R-1-R, Sequence B became 2-R-4-R-1-R-3-R. This change in sequence structure affected the occurrence probability of certain triplets (Fig. 2B). Specifically, 75% of triplets classified as high-probability during the Learning Phase became low-probability in the Rewiring Phase (HL, with the first letter denoting the triplet probability in Sequence A and the second letter referring to the probability in Sequence B). Conversely, new high-probability triplets (LH) emerged during the Rewiring Phase, replacing those that became low-probability. Meanwhile, the probability of other triplets remained constant, either maintaining a low probability (LL) or a high probability (HH) in both phases.

The LH triplets were crucial for assessing the acquisition of new knowledge during the Rewiring Phase. As these triplets transitioned from low- to high-probability in the Rewiring Phase, any associated knowledge could be considered acquired during this session. The LL triplets, on the other hand, served as a baseline for controlling general practice effects. Thus, the reaction time difference between LH and LL triplets was used as a key measure of participants’ rewiring ability in the second session^15^.

##### Session 3: retrieval phase

During the Retrieval Phase, participants completed 30 blocks of the ASRT task. Unbeknownst to them, they were tested on both Sequence A and Sequence B. To ensure balanced testing, half of the participants started with Sequence A followed by Sequence B, while the other half started with Sequence B followed by Sequence A. Participants completed five blocks of one sequence before switching to the other sequence, and this alternation continued until all participants completed 15 blocks of each sequence (i.e., 30 blocks in total). Knowledge of Sequence A was assessed by computing the difference between LL and HL triplets, whereas knowledge of Sequence B was evaluated by comparing LL and LH triplets^15^.

#### Measures of executive functions (Session 4)

The assessment of EF was guided by the Unity and Diversity model of EF, which encompasses inhibition, updating, and shifting processes^47,48^. Additionally, we examined executive control of attention, working memory, and verbal fluency, which are also prefrontal-dependent functions^59,78–82^. To operationalize these constructs, we employed a battery of well-established and reliable cognitive tests, which included the Attentional Network Test, the Berg Card Sorting Test, the Counting Span Task, the Go/No-Go Task, and three verbal fluency tasks. These tests were selected based on their well-documented validity and reliability in previous research^17,83–87^, ensuring robust measurement of the targeted cognitive functions.

#### Attentional network test (ANT)

The ANT assessed three distinct attention networks: the alerting, orienting, and executive networks^88^. Participants had to identify whether a central arrow, embedded in an array of five arrows, pointed left or right. The array could appear above or below a fixation cross and might be preceded by a spatial cue indicating the subsequent location. Additionally, in half of trials, the central arrow was congruent with the other arrows (pointing in the same direction), and in the other half it could be incongruent with the other arrows (pointing in the opposite direction).

To compute network scores, we adhered to the standard methodology^88^. The alerting component was derived by subtracting the mean reaction time (RT) of the central cue conditions from the mean RT of the no-cue conditions for each participant. The orienting component was computed by subtracting the mean RT of the spatial cue conditions from the mean RT of the central cue conditions. The executive component of attention was determined by subtracting the mean RT of all congruent conditions from the mean RT of all incongruent conditions. In this task, higher scores across the alerting, orienting, and executive domains indicated enhanced performance in the respective aspects of attention. In simpler terms, elevated scores suggest better attentional abilities in these three facets of attentional processing.

#### Berg card sorting task (BCST)

We assessed set shifting or cognitive flexibility using the computerized version of the Berg Card Sorting Test^89^ with 64 cards (BCST.64), available in the Psychology Experiment Building Language (PEBL) software^90^. In this test, a set of four cards with varying characteristics—color, shape, and number of items—was displayed at the top of the screen. Participants were instructed to match new cards to those at the top based on one of the three characteristics, without knowing the current rule but received feedback after each attempt. The rule could change during the task. Cognitive flexibility was measured through perseverative errors, which indicated failure to adapt quickly after a rule change. In essence, these errors provide insights into the participant’s ability to adjust their cognitive approach when the task requirements change.

#### Counting span (CSPAN) task

To assess updating or working memory capacity, we used the CSPAN task^91^. In this task, various shapes, including blue circles, blue squares, and yellow circles, were presented on a computer screen. Participants were instructed to audibly count and remember the number of blue circles (targets) among other shapes (distractors) in a series of images. Each image consisted of three to nine blue circles, one to nine blue squares, and one to five yellow circles. After each trial, participants verbally reported the total number of targets in the image. If the count was accurate, the experimenter proceeded to the next trial. A recall cue at the end of a set prompted participants to recall the number of targets for each image in their order of presentation. If the recall was correct, a new set with an additional image commenced, up to maximum six images in a set. If a participant made an error in recall, the task was halted, and a new run, starting with a set of two images, commenced. Each participant completed three runs of the task. Memory span was computed as the mean of the highest set sizes that the participant correctly recalled in the three runs. This measure provides an insight into the participant’s ability to efficiently update and maintain information in working memory.

#### Go/No-Go (GNG) task

We measured cognitive inhibition with the computerized version of the GNG task available in the PEBL software^90^. In the GNG task, participants were instructed to respond to certain stimuli (“go” stimuli) by clicking on a button as fast as possible and to refrain from clicking on other stimuli (“no-go” stimuli). In this version, participants were presented with a 2 × 2 array with four blue stars (one in the center of each square of the array). Every 1500 ms, a stimulus (the letter P or R) would appear for 500 ms in the place of one of the blue stars. For the first half of the task, the letter P would be the “go” stimulus and the letter R would be the “no-go”. This rule would be then inverted in the second half of the task. Participants completed 320 trials. The ratio between “go” and “no-go” trials was 80:20, respectively. Cognitive inhibition capacity in the GNG task was measured by the d’:

$$d\prime ={\text{ }}Z\left( {hit{\text{ }}rate} \right){\text{ }} - {\text{ }}Z\left( {false{\text{ }}alarm{\text{ }}rate} \right),$$

with higher *d’* scores indicating better cognitive inhibition.

#### Verbal fluency tasks

Verbal fluency was assessed through three subtasks, examining the lexical, semantic, and action components of verbal fluency. In the lexical fluency subtask, participants were tasked with generating as many words as possible beginning with the letter “P,” a common practice in the French version of phonemic fluency^92^. For the semantic fluency subtask, participants were instructed to name animals, and for the action fluency subtask, they had to articulate isolated verbs describing actions feasible for a person to perform.

In each subtask, participants aimed to produce as many words as possible within one minute while adhering to specific guidelines: avoiding word repetitions, words with the same etymological root, and proper nouns. Any violation of these rules constituted an error. The score for each verbal fluency subtask was calculated by subtracting the number of errors from the total words generated within the one-minute timeframe. A higher score in each verbal fluency component denoted greater verbal fluency capacity, reflecting the participant’s ability to generate words efficiently while adhering to specified constraints.

### Experimental design

Participants took part in a four-session experiment (Fig. 2A). The first three sessions were dedicated to assessing the acquisition, rewiring, and retrieval of implicit probabilistic information, each separated by a 24-hour interval. In the initial session (Learning Phase), participants’ SL abilities were evaluated using the ASRT task. Here, participants acquired associations related to Sequence A. In the second session (Rewiring Phase), a structural change was introduced to the task by incorporating Sequence B (see Tasks section for details). This modification allowed us to measure participants’ rewiring abilities. In the third session (Retrieval Phase), participants were tested on both sequences A and B in a counterbalanced order, providing insight into the consolidation of initial and rewired knowledge. Half of the participants started the Retrieval Phase with Sequence A and the other half with Sequence B. The fourth session was conducted independently from the first three sessions and was planned according to each participant’s availability. In this session, prefrontal-dependent cognitive functions were assessed with a battery of well-established and reliable neuropsychological tasks: the ANT, the BCST, the CSPAN task, the GNG task and three variants of the Verbal Fluency tasks (action, lexical and semantic fluency).

### Statistical analysis

Evaluating someone’s ability to rewire their knowledge during the Rewiring Phase requires that the initial knowledge is acquired during the Learning Phase. Therefore, participants who did not exhibit initial SL during the Learning Phase (i.e., L-H < 0) were excluded. This refinement in participant selection aimed to enhance the relevance and meaningfulness of all subsequent analyses focusing on the relationship between rewiring and EF. Seventeen participants did not demonstrate SL during the Learning Phase. Consequently, our analyses were performed using the data of the remaining 59 participants.

#### Learning, rewiring and retrieval trajectories

For the Learning, Rewiring, and Retrieval Phases, the first five trials (comprising five warm-up random trials) of each block were excluded from the analysis, along with all incorrect responses throughout all trials. Additionally, trials featuring trills (e.g., 2-1-2) or repetitions (e.g., 2-2-2) were omitted from the analysis because participants tend to respond more rapidly to these types of triplets due to pre-existing tendencies^94^. To facilitate the analysis and improve the signal-to-noise ratio, the blocks of the ASRT task were organized into units of five blocks, referred to as bins.

In the first session (Learning Phase), learning performance on Sequence A was assessed by calculating the median RTs within each bin for correct responses in high-probability triplets specific to Sequence A (HL) and low-probability triplets common to all sessions (LL), which served as a baseline to control for general practice effects. Subsequently, learning scores were computed for each bin by subtracting the median RTs of HL triplets from the median RTs of LL triplets. A higher difference score between HL and LL triplets indicated better initial learning of probabilities. The learning scores for each bin were then averaged to generate a single score reflecting statistical learning (SL) for each participant in the Learning Phase.

In the second session (Rewiring Phase), rewiring performance on Sequence B was evaluated by calculating the median RTs for correct responses in high-probability triplets specific to Sequence B (LH) and LL triplets. Data processing followed a similar approach to the first session, with the unique distinction being that rewiring scores resulted from the difference between LH and LL triplets. A higher difference score between LH and LL triplets indicated better rewiring performance.

During the third session (Retrieval Phase), participants alternated between bins of sequences A and B in a counterbalanced order. Data for assessing the retrieval of Sequence A was processed similarly to the Learning Phase, while data for Sequence B retrieval followed the same procedure as in the Rewiring Phase.

To evaluate SL in the Learning and Rewiring Phases separately, we conducted a repeated measures analyses of variance (RM ANOVAs) with two factors for each phase: bin (1–5) and triplet (HL vs. LL for the Learning Phase; LH vs. LL for the Rewiring Phase). To compare the retrieval of Sequences A and B in the third session, we conducted an RM ANOVA with two factors: bin (1–3; corresponding to the three bins presented for each sequence in the Retriaval Phase), and sequence (A vs. B). In all ANOVAs, Greenhouse-Geisser epsilon correction was used when Mauchly’s test of sphericity indicated that sphericity could not be assumed. Original df values and p values are reported together with partial eta-squared (η_p_^2^) as the measure of effect size.

To complement these analyses, we conducted Bayesian RM ANOVAs using JASP^93^. Bayesian analysis allows us to provide evidence in favor of the null hypothesis, rather than failing to reject it. The inclusion Bayes factor quantifies the change from prior inclusion odds to posterior inclusion odds and can be interpreted as evidence in the data for including a predictor or factor. More generally, Bayes factors quantify the relative weight of evidence provided by the data for two theories, the null and the alternative hypotheses, H0 and H1^95^. According to a commonly used classification^96^, BF values between 1 and 3 indicate anecdotal evidence, values between 3 and 10 indicate substantial evidence, and values above 10 indicate strong evidence for H1. Conversely, values between 1/3 and 1 indicate anecdotal evidence, values between 1/10 and 1/3 indicate substantial evidence, and values below 1/10 indicate strong evidence for H0. Values near 1 indicate that the data do not favor either hypothesis.

#### Correlations

For our correlation and multiple regression analyses, we derived single indices of learning, rewiring, and retrieval of sequences A and B by averaging scores across all bins for each participant. Prior research has indicated that while the reliability of individual bins is typically low, averaging across at least five bins results in acceptable levels of reliability^72^. Given the importance of using reliable measures for assessing inter-individual differences, our analysis of the relationship between rewiring and EF utilized these average scores. Our reported Pearson’s correlations between learning, rewiring, retrieval and EF scores include Benjamin-Hochberg adjusted p-values to account for multiple comparisons. Additionally, we calculated Bayes factors using JASP^93^ with default priors (stretched beta distribution with a width of 1) to further validate our findings.

#### Multiple regression analysis

To assess the relationship between EF and the different phases of the experiment (i.e., learning, rewiring, and retrieval), we conducted multiple linear regression analyses. The predictor variables included the scores of the executive component of the ANT, BCST, CSPAN task, GNG task, and verbal fluency tasks. Note that the alerting and orienting components of the ANT were not included in the models as these do not reflect EF^88^. The regression analyses were applied systematically to the outcome variables for each experimental phase.

For the Learning Phase, the EF tests were used as predictor variables to assess their contribution to the variance in the initial learning performance on (Sequence A). For the Rewiring Phase, we explored the association between EF test scores and rewiring performance (on Sequence B). For the Retrieval Phase, two separate multiple regression analyses were conducted to assess the relationship between EF and the retrieval of Sequence A and Sequence B. Multicollinearity among predictor variables was assessed using variance inflation factor (VIF) in each model. All VIF values were below the commonly accepted threshold of 5, with the highest value being 1.804 for action fluency, indicating that multicollinearity is not a concern in our models.

## Results

### Learning, rewiring and retrieval trajectories

#### Learning phase

**Fig. 3:**
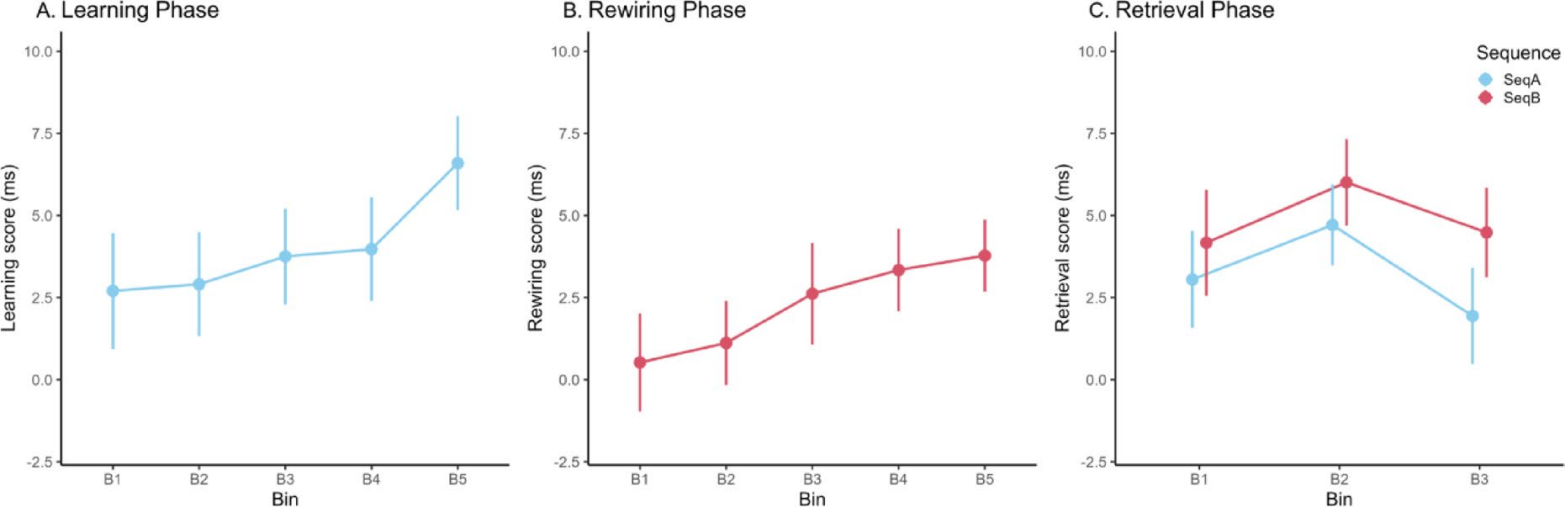
Acquisition and retrieval of implicit knowledge during the different phases of the experiment. (**A**) Leaning Phase. Acquisition of the initial sequence during the Learning Phase. The learning scores for each bin denote the difference of RTs between HL and LL triplets. Participants acquired the probabilistic regularities of Sequence A, responding faster to HL triplets compared to LL triplets. (**B**) Rewiring Phase. Acquisition of a new sequence during the Rewiring Phase. The rewiring scores for each bin denote the difference of RTs between LL and LH triplets. Participants acquired the probabilistic regularities of Sequence B, responding faster to LH triplets compared to LL triplets. (**C**) Retrieval Phase. Retrieval of both sequences during the Retrieval Phase. The retrieval scores denote the differences between HL and LL triplets for Sequence A (in blue), and LH and LL triplets for Sequence B (in red). Sequence A and Sequence B knowledge was retrieved equivalently and did not vary across the bins. Error bars in all figures denote the standard error of the mean.

To evaluate SL in the learning phase, we conducted a repeated measures analysis of variance (RM ANOVA) with two factors: bin (1–5) and triplet (HL vs. LL). The main effect of bin was significant (*F*_(4, 232)_ = 13.056, *p* <.001, η_p_^2^ = 0.184, BF_inclusion_ = 3.527e + 9), indicating general skill learning, i.e., participants became faster overall as the task progressed, irrespective of triplets (bin 1 significantly slower than all further bins all *p*-values < 0.001, all other pairwise comparisons *p* >.248). The main effect of triplet was significant (*F*_(1, 58)_ = 12.395, *p* <.001, η_p_^2^ = 0.176, BF_inclusion_ = 14816.224), with average responses to HL triplets being faster than responses to LL triplets (M_HL_ = 358 ms, 95% CI = [352, 364]; M_LL_ = 362 ms, 95% CI = [356, 368]; d = 0.157), revealing successful SL during the Learning Phase (Fig. 3A). The bin by triplet interaction was non-significant (*F*_(4, 232)_ = 1.644, *p* =.164, η_p_^2^ = 0.028, BF_inclusion_ = 0.121) indicating that SL did not change substantially across the bins.

#### Rewiring phase

To evaluate SL in the Rewiring Phase, we conducted an RM ANOVA with two factors: bin (1–5) and triplet (LH vs. LL). The main effect of bin was non-significant (*F*_(4, 232)_ = 0.513, *p* =.726, η_p_^2^ = 0.009, BF_inclusion_ = 0.006), indicating that, irrespective of triplets, the RTs did not progress in the second session. Importantly, the main effect of triplet was significant (*F*_(1, 58)_ = 5.596, *p* =.021, η_p_^2^ = 0.088, BF_inclusion_ = 12.602), with average responses to LH triplets being faster than responses to LL triplets (M_LH_ = 347 ms, 95% CI = [341, 353]; M_LL_ = 349 ms, 95% CI = [344, 355]; d = 0.098), indicating updating of the initial knowledge during the second session (Fig. 3B). The bin by triplet interaction was non-significant (*F*_(4, 232)_ = 1.797, *p* =.130, η_p_^2^ = 0.030, BF_inclusion_ = 0.001), indicating that rewiring performance did not vary substantially across the bins.

#### Retrieval phase

To evaluate the retrieval of sequences A and B in the third session, we conducted an RM ANOVA, this time on the learning scores, with two factors: bin (1–3) and sequence (A vs. B). Learning scores for both sequences were significantly different from 0 in almost all bins (*p*s < .043), except the Sequence A learning score for bin 3, which did not significantly differ from 0 (*p* =.190). The main effect of bin was non-significant (*F*_(2, 116)_ = 2.081, *p* =.129, η_p_^2^ = 0.035, BF_inclusion_ = 0.168) indicating that learning scores remained relatively constant during the retrieval phase. The main effect of sequence was non-significant (*F*_(1, 58)_ = 3.220, *p* =.078, η_p_^2^ = 0.053, BF_inclusion_ = 0.421) indicating that both sequences were retrieved equally during the third session (Fig. 3C), although there is a trend towards smaller Sequence A learning scores (M_SeqA_ = 3.23 ms, 95% CI = [1.02, 5.45]; M_SeqB_ = 4.89 ms, 95% CI = [2.63, 7.15]; d = 0.152). The bin by sequence interaction was non-significant (*F*_(2, 116)_ = 0.300, *p* =.742, η_p_^2^ = 0.005, BF_inclusion_ = 0.022) indicating that the retrieval of sequences A and B did not vary across the bins.

### Correlations

**Fig. 4:**
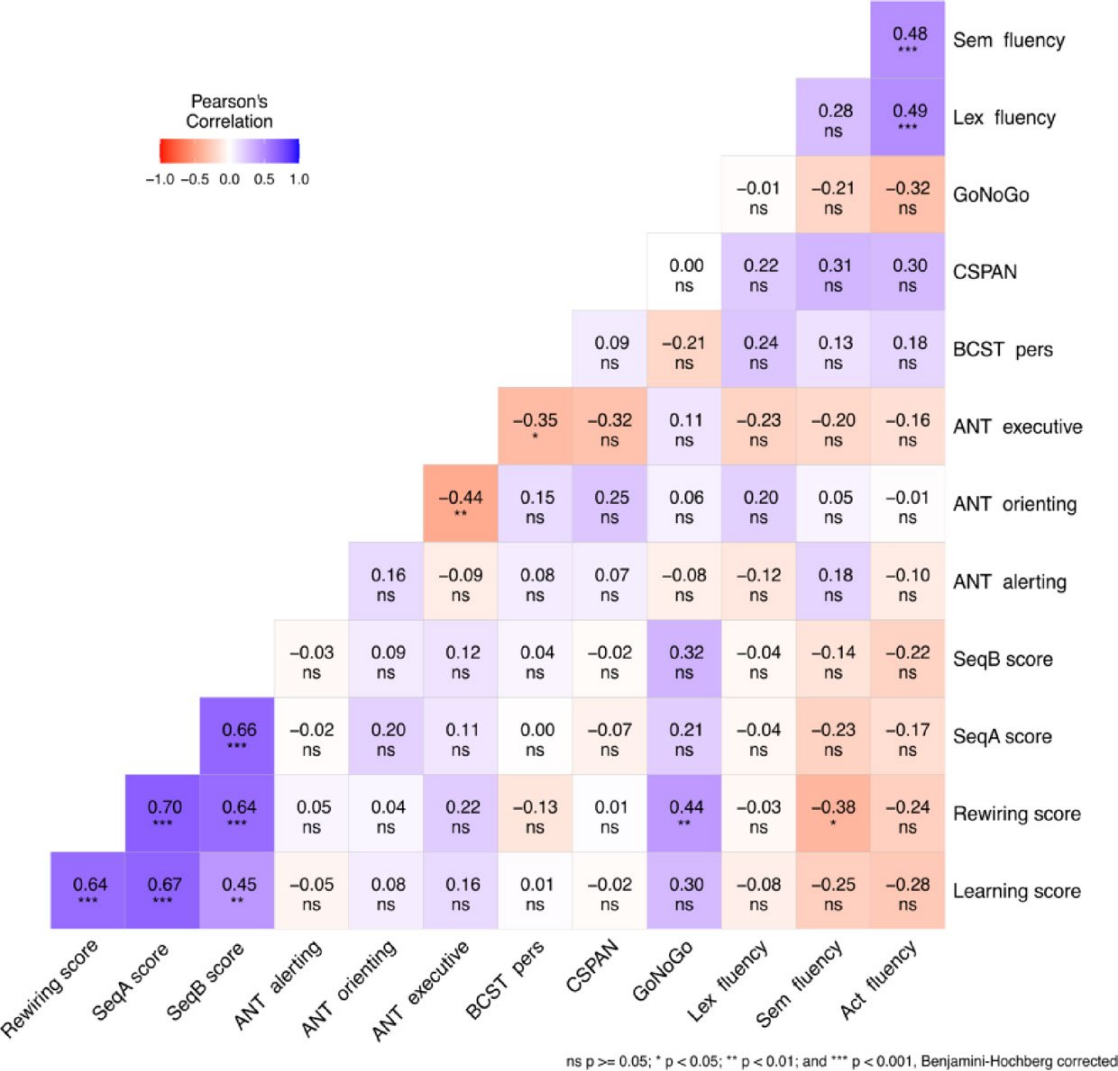
Correlations Between Learning, Rewiring, Retrieval Scores, and EF Measures. Negative and positive correlations are visually represented by red and blue backgrounds, respectively. A significant positive correlation was found between cognitive inhibition (measured by GNG) and rewiring performance, while a significant negative correlation was found between semantic fluency and rewiring. Benjamin-Hochberg corrected p-values are included in the matrix. The Learning score reflects knowledge acquired in Session 1, while the Rewiring score reflects the updating of knowledge (i.e., rewiring performance) in Session 2. SeqA score indicates the retrieval of initial knowledge in Session 3, and SeqB score reflects the retrieval of updated knowledge in Session 3. ANT alerting, orienting, and executive represent the three aspects of the Attentional Network Test. BCST pers score denotes cognitive flexibility performance in the Berg Card Sorting Test. CSPAN indicates working memory performance in the Counting Span task. Go/No-Go reflects inhibition performance in the Go/No-Go task. Lastly, Act, Sem, and Lex fluency scores represent performances in the Action Fluency, Semantic Fluency, and Lexical Fluency tasks, respectively.

Bivariate Pearson’s correlations with Benjamin-Hochberg adjusted p-values between all variables are presented in Fig. 4. Overall, most EF measures showed weak or non-significant correlations with learning, rewiring, and retrieval scores for sequences A and B. The strongest correlations were found between the rewiring and GNG scores (Pearson’s *r* =.439, 95% CI = [0.206, 0.625], *p* = .003, BF_10_ = 59.484) and between the rewiring and Semantic fluency scores (Pearson’s *r* = −.378, 95% CI = [−0.578, − 0.135], *p* = .015, BF_10_ = 11.450).

### Multiple regression analysis

#### Rewiring phase

**Fig. 5:**
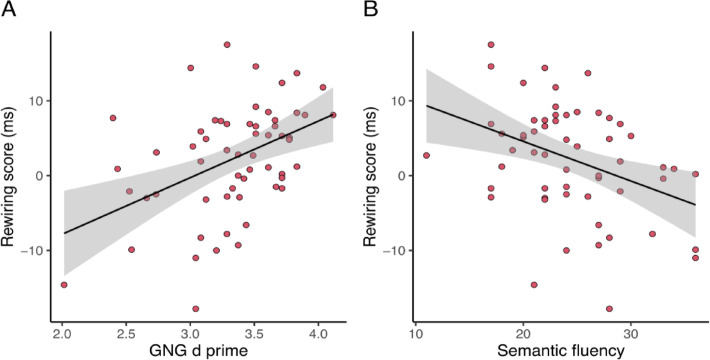
Relationship between the Rewiring score and the GNG d-prime and Semantic fluency scores. Each data point represents a participant. The black line represents the best-fit regression line, and the gray shading indicates the 95% confidence interval.

To test whether EF scores predict the rewiring of implicit probabilistic information, a multiple regression analysis was conducted with the rewiring score as the dependent variable. Predictor variables included the executive component of ANT, BCST, CSPAN, GNG, and verbal fluency tasks (action, semantic, and lexical). Together, these predictors explained 22.6% of the variance in rewiring performance (Adjusted R^2^ = 0.226, *F*_(7, 51)_ = 3.418, *p* = .005). Notably, the GNG score emerged as a strong predictor of rewiring (B = 5.881, 95% CI = [1.458, 10.304], *p* =.010, β = 0.341), suggesting that better inhibition, as measured by GNG performance, was associated with more effective rewiring of implicit probabilistic representations (Fig. 5A). In contrast, the semantic fluency score negatively predicted rewiring performance (B = −0.455, 95% CI = [−0.838, −0.073], *p* =.021, β = − 0.325), indicating that better semantic fluency performance was inversely related to rewiring (Fig. 5B). Other EF scores, including ANT executive, BCST, CSPAN, lexical fluency, and action fluency, did not significantly contribute to the model (Supplementary Table 2).

#### Learning and retrieval phases

In line with the correlation results reported above, multiple regression analyses for learning in Session 1 and retrieval of Sequences A and B in Session 3 revealed no significant predictors. Details of these multiple regression results are presented in Supplementary Tables 3, 4, and 5, respectively.

## Discussion

When the environment changes, our old habits and predictions no longer function optimally. Our study aimed to explore how individual differences in EF affect the updating of our predictive models, leading to changes in our habits. Our findings revealed distinct correlations between EF and the rewiring of implicit probabilistic representations. Specifically, we observed a positive relationship between inhibition, measured by the Go/No-Go task, and rewiring. We also observed a negative relationship between semantic fluency and rewiring. These correlations were further confirmed by multiple regression analysis, which showed that while Go/No-Go scores positively predicted rewiring performance, semantic fluency scores negatively predicted it. This suggests that individuals with stronger inhibitory control and/or lower semantic fluency tend to exhibit a better capacity to update their probabilistic representations during rewiring.

The positive relationship between rewiring and the Go/No-Go task suggests that the ability to inhibit irrelevant or outdated information and behaviors may facilitate the adaptation to new probabilistic contingencies in the presence of pre-existing knowledge. This positive correlation resonates with existing literature on EF and habit change, where inhibitory control has been shown to play a crucial role in disrupting and modifying unhealthy habits and ingrained behaviors^97–100^ Cognitive inhibition may facilitate habit change by shielding the individual from maladaptive memory representations^101^ and suppressing automatic motor responses tied to old habits^102^. This may be particularly relevant in our task, where successful rewiring requires suppressing automatic motor responses tied to the old sequence. It is worth noting that we focused on response inhibition (Go/No-Go task) rather than interference control (e.g., Stroop task). While response inhibition appears critical for overriding learned motor actions, overcoming proactive interference from prior cognitive representations is also a key challenge in rewiring. Future research employing tasks that measure both facets of inhibition could help disentangle their respective contributions to the updating of implicit knowledge.

Intriguingly, this finding contrasts with previous research which reported that inhibitory control was detrimental to the rewiring of probabilistic representations^15^. Notably, efforts to understand the relationship between SL and EF have led to various experimental approaches, generally categorized into four main types. The first involves behavioral studies adopting an interindividual differences approach, measuring SL and EF with separate tasks and investigating the correlations between their performance indices. These studies have found both positive and negative associations, suggesting potential cooperation, competition, and instances of independence^65,67,68^. The second category includes interventional studies that deplete, disrupt, or suppress EF to observe how this affects SL, generally finding a competitive relationship, with weakened EF leading to better SL performance^61–63^. The third category focuses on brain network studies, examining the relationship between SL and the disengagement of brain networks supporting EF, and finding that such disengagement improves SL^60,65,66^. The fourth category employs combined paradigms that integrate features of both SL and EF in a single experimental design, providing direct insight into their real-time interactions. Studies following this approach have shown evidence for both competition and independence between SL and EF^103,104^.

While most of these studies focus on the initial acquisition of probabilistic knowledge, their methodological approaches can also be extended to study the relationship between rewiring and EF. Our study follows the first approach. In another study, participants actively inhibited motor responses associated with the knowledge acquired in the Learning Phase, whereas in our study, we measured interindividual differences in inhibitory control capacity^15^. This methodological difference may explain the discrepancy in results. It is possible that, in Horvath et al., individuals’ cognitive inhibitory resources were allocated to refraining from specific motor responses rather than inhibiting implicit probabilistic representations acquired in the Learning Phase. These results can be interpreted within a dual-task framework, which suggests that if a secondary task impairs performance, the cognitive function engaged in the secondary task is crucial for achieving the good performance in the primary task^105^. Thus the weaker rewiring scores observed in the previous study for inhibited (no-go) trials compared to go trials may indicate that inhibitory capacity is essential for the rewiring process itself^15^.

Conversely to the positive relationship between rewiring and inhibition observed in our study, we found a negative correlation between rewiring and semantic fluency. This result suggests that individuals with higher semantic fluency may be less proficient at updating their implicit probabilistic representations in response to environmental changes. While this result might seem unexpected within the broader context of EF and habit change research^52^, it is consistent with the competitive learning and memory systems framework^41,44,60,68,70^. Given that verbal fluency tasks, especially semantic fluency, involves the controlled access to long term memory representations^106–108^, a possible interpretation for our result could be that individuals with greater verbal fluency rely more heavily on pre-existing cognitive mnesic models, which could impede their ability to integrate novel probabilistic information^68^. This negative relationship aligns with the competition hypothesis of dual-process neuro-computational models where a greater reliance on model-based cognitive functioning is associated with a smaller reliance on model-free processes^39,42,44,109^. In this context, proficiency in semantic fluency could reflect more efficient access to long-term memory, suggesting a heavier reliance on model-based processes. This reliance may hinder the acquisition of new probabilistic information, which may be dependent on the model-free system. Similar results were also reported, suggesting that the ability to acquire new probabilistic representations might be related to a reduced reliance on model-based functioning, as reflected by poorer performance on verbal fluency tasks^68^.

It is important to note, however, that while verbal fluency tasks are generally considered good indicators of EF^110,111^, their precise role within the Unity and Diversity model of EF^47^ is still debated. Studies examining verbal fluency tasks in the context of Miyake’s model have suggested that these tasks may reflect cognitive flexibility or set shifting^110,112^. Based on this, it could be hypothesized that the negative correlation between rewiring and verbal fluency found in our study reflects competition between updating probabilistic representations and cognitive flexibility. Nonetheless, this interpretation seems unlikely given our data, as no relationship was found between rewiring and BCST, a well-established set-shifting task^86^. Additionally, the lack of correlation between BCST and verbal fluency tasks in our analysis further suggests that verbal fluency tasks are not purely measures of cognitive flexibility. Semantic, lexical, and action fluency tasks likely involve different cognitive processes and may differ to what extent they reflect EF. For example, while cognitive flexibility has been shown to be the primary factor in the lexical fluency task, the semantic fluency task may also involve other components such as categorical production^112^, which likely reflects long-term memory access^113^. These distinctions support our interpretation that semantic fluency reflects model-based functioning, particularly within the context of the competition hypothesis of dual-process neuro-computational processes.

Verbal fluency tasks have also been argued to reflect working memory updating^114–116^. In the context of our results, this could imply that working memory updating proficiency might counteract the rewiring of probabilistic representations. However, this interpretation seems unlikely given our data, as we observed no relationship between the Counting Span task, a valid and reliable working memory task^117^, and rewiring or verbal fluency. In our previous study^68^, using exploratory factor analysis we have identified a common factor underlying both the Counting Span and verbal fluency tasks, which negatively correlated with SL. We have suggested that this factor could reflect either updating or access to long-term memory models. Based on our current results, the latter explanation seems more plausible. This interpretation aligns with results that suggest that the updating of implicit probabilistic representations depends exclusively on model-free processes^37^. It is also important to note that the updating referenced in the context of EF models differs from the updating involved in the probabilistic learning and neuro-computational modeling framework. The former involves the conscious maintenance and manipulation of information in working memory^48,118^, whereas the latter involves the implicit modification of informational priors^119,120^. This is consistent with our interpretation that not the updating in working memory, but the access to long-term memory affects rewiring performance.

Our correlational analyses results were further corroborated through multiple regression analysis, which revealed that inhibition, measured by the Go/No-Go task, positively predicted rewiring performance, while semantic fluency negatively predicted rewiring it. This statistical approach strengthened the validity of our results and provided additional support for the observed relationships between executive functions and the rewiring of probabilistic representations^121^. Our findings highlight the complex cognitive dynamics involved in the rewiring of implicit probabilistic representations. Successful rewiring appears to require a balance between inhibiting outdated cognitive schemas and acquiring new probabilistic information. Different cognitive functions, such as inhibition and verbal fluency, may contribute to these processes in distinct ways. Inhibition may facilitate the suppression of motor responses and memory representations related to outdated knowledge, facilitating the integration of new information^101,102^. Conversely, verbal fluency may reflect a greater reliance on model-based functioning, which could hinder the flexibility needed to acquire novel probabilistic representations^44,68,107,108^.

These findings carry significant theoretical implications for understanding the cognitive mechanisms involved in acquiring probabilistic information across various contexts. In the literature, there has been ongoing discourse regarding whether the acquisition of new information, whether under conditions of proactive interference or not, relies on similar or distinct neural and cognitive mechanisms^15,122,123^. The negative correlation we observed between semantic fluency and rewiring performance echoes findings from studies investigating the link between EF and learning^67,68^. This suggests a possible convergence in the neurocognitive mechanisms that support both learning and rewiring processes. Nonetheless, this hypothesis warrants further investigation, particularly through studies exploring the cellular and neural architectural dynamics underlying learning and rewiring of probabilistic information.

Our findings also highlight the potential for cognitive control interventions, particularly those targeting inhibitory control, to enhance the rewiring process. Research in fields such as food intake and addiction has shown that cognitive control training can effectively modify habitual behaviors^52,97,98^. Bolstering inhibitory control could enable individuals to better suppress outdated cognitive models and more easily integrate new information, facilitating adaptive learning and habit updating. Nonetheless, it is important to acknowledge the limitations of this study. For instance, the Rewiring Phase and executive function measurements were conducted on separate days. Intraindividual differences in the reliance on model-based or model-free functioning may be influenced by factors such as emotional state^124,125^ or hormonal fluctuations during the menstrual cycle^126,127^. These factors could contribute to variability in our findings. While other approaches such as Canonical Correlation Analysis could reveal powerful associations between our multivariate executive function and ASRT measures, offering deeper insights into how these distinct cognitive processes interrelate, our current sample size is too small for reliable results with this method. Furthermore, future studies could explore alternative metrics of learning beyond average performance, such as the rate of acquisition (i.e., the learning slope) or asymptotic performance levels, which may capture different aspects of learning dynamics and relate differently to executive functions. Future studies should address these limitations to improve the robustness and generalizability of the results. Additionally, incorporating neuroimaging techniques such as fMRI or EEG could elucidate the neural mechanisms underlying the interplay between these cognitive processes.

A further limitation of our study is the relatively small sample size (*N* = 59) used for the main analyses, which resulted from the necessary exclusion of participants who did not show evidence of initial learning. This sample size may limit the statistical power of our analyses and the stability of the correlation estimates. For instance, we found a specific negative relationship between rewiring and semantic fluency, but not lexical or action fluency, even though these tasks were inter-correlated. While we have offered a theoretical interpretation for this specificity, it is possible that this pattern is an artifact of our sample size. Therefore, our findings, particularly the nuanced relationships with specific EF measures, should be interpreted with caution and warrant replication in larger, more powered studies to ensure their robustness and generalizability.

To our knowledge, this study is the first to explore the relationship between the rewiring of implicit probabilistic information and a wide range of prefrontal-dependent executive functions. Our findings suggests a nuanced interaction between these cognitive processes, as revealed through the study of individual differences: while rewiring showed a positive relationship with cognitive inhibition, it was negatively associated with verbal fluency. This indicates that the rewiring of implicit probabilistic representations is a multifaceted cognitive process intricately intertwined with executive functions. It appears to necessitate both overcoming proactive interference from prior knowledge, facilitated by cognitive inhibition, and a reliance on model-free functioning to integrate novel probabilistic information, as evidenced by the negative relationship with semantic fluency. By shedding light on the relationship between executive functions and rewiring, this study provides a foundational understanding of the cognitive dynamics underlying habit change.

## Supplementary Information

Below is the link to the electronic supplementary material.

Supplementary Material 1

## Data Availability

Code and data necessary to replicate these findings can be found at the following link: https://osf.io/gqpjn/.

## References

[1] Poldrack, R. A. *Hard To Break: why our Brains Make Habits Stick* (Princeton University Press, 2021).

[2] Dickinson, A. Actions and habits: the development of behavioural autonomy. Philosophical transactions of the Royal society of London B. *Biol. Sci.* **308**, 67–78. 10.1098/RSTB.1985.0010 (1985).

[3] Dickinson, A. & Balleine, B. Motivational control of goal-directed action. *Anim. Learn. Behav.* **22**, 1–18. 10.3758/BF03199951/METRICS (1994).

[4] Foerde, K. What are habits and do they depend on the striatum? A view from the study of neuropsychological populations. *Curr. Opin. Behav. Sci.* **20**, 17–24. 10.1016/J.COBEHA.2017.08.011 (2018).

[5] Seger, C. A. & Spiering, B. J. A critical review of habit learning and the basal ganglia. *Front. Syst. Neurosci.* **5**, 9748. 10.3389/FNSYS.2011.00066/BIBTEX (2011). 10.3389/fnsys.2011.00066 PMC3163829 21909324

[6] Wood, W. & Rünger, D. Psychology of habit. *Annu. Rev. Psychol.* **67**, 289–314. 10.1146/annurev-psych-122414-033417 (2016). 26361052 10.1146/annurev-psych-122414-033417

[7] Carden, L. & Wood, W. Habit formation and change. *Curr. Opin. Behav. Sci.* **20**, 117–122. 10.1016/J.COBEHA.2017.12.009 (2018).

[8] Logan, G. D. Toward an instance theory of automatization. *Psychol. Rev.* **95**, 492–527. 10.1037/0033-295X.95.4.492 (1988).

[9] Hagger, M. S., Cameron, L. D., Hamilton, K., Hankonen, N. & Lintunen, T. *The Handbook of Behavior Change* (Cambridge University Press, 2020).

[10] Verplanken, B. & Roy, D. Empowering interventions to promote sustainable lifestyles: testing the habit discontinuity hypothesis in a field experiment. *J. Environ. Psychol.* **45**, 127–134. 10.1016/J.JENVP.2015.11.008 (2016).

[11] Verplanken, B., Walker, I., Davis, A. & Jurasek, M. Context change and travel mode choice: combining the habit discontinuity and self-activation hypotheses. *J. Environ. Psychol.* **28**, 121–127. 10.1016/J.JENVP.2007.10.005 (2008).

[12] Wood, W., Tam, L. & Witt, M. G. Changing circumstances, disrupting habits. *J. Pers. Soc. Psychol.* **88**, 918–933. 10.1037/0022-3514.88.6.918 (2005). 15982113 10.1037/0022-3514.88.6.918

[13] Graybiel, A. M. Habits, rituals, and the evaluative brain. *Annu. Rev. Neurosci.* **31**, 359–387. 10.1146/ANNUREV.NEURO.29.051605.112851/CITE/REFWORKS (2008). 18558860 10.1146/annurev.neuro.29.051605.112851

[14] Szegedi-Hallgató, E. et al. Explicit instructions and consolidation promote rewiring of automatic behaviors in the human Mind. *Sci. Rep.* **7**, 4365. 10.1038/s41598-017-04500-3 (2017). 28663547 10.1038/s41598-017-04500-3 PMC5491510

[15] Horváth, K., Nemeth, D. & Janacsek, K. Inhibitory control hinders habit change. *Sci. Rep.* **12**, 1–11. 10.1038/s41598-022-11971-6 (2022). 35585209 10.1038/s41598-022-11971-6 PMC9117234

[16] Aslin, R. N. Statistical learning: a powerful mechanism that operates by Mere exposure. *Wiley Interdiscip Rev. Cogn. Sci.* 8. 10.1002/wcs.1373 (2017). 10.1002/wcs.1373 PMC5182173 27906526

[17] Conway, C. M. How does the brain learn environmental structure? Ten core principles for Understanding the neurocognitive mechanisms of statistical learning. *Neurosci. Biobehav Rev.* **112**, 279–299. 10.1016/j.neubiorev.2020.01.032 (2020). 32018038 10.1016/j.neubiorev.2020.01.032 PMC7211144

[18] Kaufman, S. B. et al. Implicit learning as an ability. *Cognition* **116**, 321–340. 10.1016/j.cognition.2010.05.011 (2010). 20573341 10.1016/j.cognition.2010.05.011

[19] Isbilen, E. S. & Christiansen, M. H. Statistical learning of language: A Meta-Analysis into 25 years of research. *Cogn. Sci.* **46**, e13198. 10.1111/COGS.13198 (2022). 36121309 10.1111/cogs.13198

[20] Saffran, J. R., Aslin, R. N. & Newport, E. L. Statistical learning by 8-month-old infants. Science 1996;274:1926–8. (1979). 10.1126/science.274.5294.1926 10.1126/science.274.5294.1926 8943209

[21] Ullman, M. T., Earle, F. S., Walenski, M. & Janacsek, K. The neurocognition of developmental disorders of Language. *Annu. Rev. Psychol.* **71**, 389–417. 10.1146/annurev-psych-122216-011555 (2020). 31337273 10.1146/annurev-psych-122216-011555

[22] Hallgató, E., Gyori-Dani, D., Pekár, J., Janacsek, K. & Nemeth, D. The differential consolidation of perceptual and motor learning in skill acquisition. *Cortex* **49**, 1073–1081. 10.1016/j.cortex.2012.01.002 (2013). 22325422 10.1016/j.cortex.2012.01.002

[23] Verburgh, L., Scherder, E. J. A., van Lange, P. A. M. & Oosterlaan, J. The key to success in elite athletes? Explicit and implicit motor learning in youth elite and non-elite soccer players. *J. Sports Sci.* **34**, 1782–1790. 10.1080/02640414.2015.1137344 (2016). 26788666 10.1080/02640414.2015.1137344

[24] Rohrmeier, M. & Rebuschat, P. Implicit learning and acquisition of music. *Top. Cogn. Sci.* **4**, 525–553. 10.1111/J.1756-8765.2012.01223 (2012). 23060126 10.1111/j.1756-8765.2012.01223.x

[25] Romano Bergstrom, J. C., Howard, J. H. & Howard, D. V. Enhanced implicit sequence learning in College-age video game players and musicians. *Appl. Cogn. Psychol.* **26**, 91–96. 10.1002/ACP.1800 (2012).

[26] Tillmann, B. & McAdams, S. Implicit learning of musical timbre sequences: statistical regularities confronted with acoustical (dis)similarities. *J. Exp. Psychol. Learn. Mem. Cogn.* **30**, 1131–1142. 10.1037/0278-7393.30.5.1131 (2004). 15355141 10.1037/0278-7393.30.5.1131

[27] Baldwin, D., Andersson, A., Saffran, J. & Meyer, M. Segmenting dynamic human action via statistical structure. *Cognition* **106**, 1382–1407. 10.1016/J.COGNITION.2007.07.005 (2008). 18035346 10.1016/j.cognition.2007.07.005

[28] Parks, K. M. A., Griffith, L. A., Armstrong, N. B. & Stevenson, R. A. Statistical learning and social competency: the mediating role of Language. *Sci. Rep.* **10**, 1–15. 10.1038/S41598-020-61047-6 (2020). 32132635 10.1038/s41598-020-61047-6 PMC7055309

[29] Ruffman, T., Taumoepeau, M. & Perkins, C. Statistical learning as a basis for social Understanding in children. *Br. J. Dev. Psychol.* **30**, 87–104. 10.1111/J.2044-835X.2011.02045.X (2012). 22429035 10.1111/j.2044-835X.2011.02045.x

[30] Hong, I., Kim, M. S. & Jeong, S. K. Flexibility and stability of habit learning depend on Temporal signal variation. *J. Exp. Psychol. Learn. Mem. Cogn.* **48**, 1–12. 10.1037/XLM0001113 (2022). 35073135 10.1037/xlm0001113

[31] Jiang, Y. V. & Sisk, C. A. Habit-like attention. *Curr. Opin. Psychol.* **29**, 65–70. 10.1016/J.COPSYC.2018.11.014 (2019). 30537679 10.1016/j.copsyc.2018.11.014

[32] Tuhkanen, S. et al. Humans use predictive gaze strategies to target waypoints for steering. *Sci. Rep.* **9**, 1–18. 10.1038/S41598-019-44723-0 (2019). 31171850 10.1038/s41598-019-44723-0 PMC6554351

[33] Thompson, C. & Sabik, M. Allocation of attention in familiar and unfamiliar traffic scenarios. *Transp. Res. Part. F Traffic Psychol. Behav.* **55**, 188–198. 10.1016/J.TRF.2018.03.006 (2018).

[34] Dresp-Langley, B. Why the Brain Knows More than We Do: Non-Conscious Representations and Their Role in the Construction of Conscious Experience. Brain Sciences. Vol 2, Pages 1–21 2011;2:1–21. (2012). 10.3390/BRAINSCI2010001 10.3390/brainsci2010001 PMC4061785 24962683

[35] Kobor, A., Janacsek, K., Takacs, A. & Nemeth, D. Statistical learning leads to persistent memory: evidence for one-year consolidation. *Sci. Rep.* **7**10.1038/s41598-017-00807-3 (2017). 10.1038/s41598-017-00807-3 PMC5429700 28396586

[36] Goedert, K. M. & Willingham, D. B. Patterns of interference in sequence learning and Prism adaptation inconsistent with the consolidation hypothesis. *Learn. Memory*. **9**, 279–292. 10.1101/lm.50102 (2002). 10.1101/lm.50102 PMC187137 12359837

[37] Kurdi, B., Gershman, S. J. & Banaji, M. R. Model-free and model-based learning processes in the updating of explicit and implicit evaluations. *Proc. Natl. Acad. Sci. U S A*. **116**, 6035–6044. 10.1073/PNAS.1820238116 (2019). 30862738 10.1073/pnas.1820238116 PMC6442571

[38] Beierholm, U. R., Anen, C., Quartz, S. & Bossaerts, P. Separate encoding of model-based and model-free valuations in the human brain. *Neuroimage* **58**, 955–962. 10.1016/j.neuroimage.2011.06.071 (2011). 21757014 10.1016/j.neuroimage.2011.06.071

[39] Daw, N. D., Niv, Y. & Dayan, P. Uncertainty-based competition between prefrontal and dorsolateral striatal systems for behavioral control. *Nat. Neurosci.* **8**, 1704–1711. 10.1038/nn1560 (2005). 16286932 10.1038/nn1560

[40] Doyon, J. et al. Contribution of night and day sleep vs. simple passage of time to the consolidation of motor sequence and visuomotor adaptation learning. *Exp. Brain Res.* **195**, 15–26. 10.1007/s00221-009-1748-y (2009). 19277618 10.1007/s00221-009-1748-y PMC2752878

[41] Lee, S. W., Shimojo, S. & O’Doherty, J. P. Neural computations underlying arbitration between model-based and model-free learning. *Neuron* **81**, 687–699. 10.1016/j.neuron.2013.11.028 (2014). 24507199 10.1016/j.neuron.2013.11.028 PMC3968946

[42] Poldrack, R. A. & Packard, M. G. Competition among multiple memory systems: converging evidence from animal and human brain studies. *Neuropsychologia* **41**, 245–251. 10.1016/S0028-3932(02)00157-4 (2003). 12457750 10.1016/s0028-3932(02)00157-4

[43] Decker, J. H., Otto, A. R., Daw, N. D. & Hartley, C. A. From creatures of habit to goal-directed learners: tracking the developmental emergence of model-based reinforcement learning. *Psychol. Sci.* **27**, 848–858. 10.1177/0956797616639301 (2016). 27084852 10.1177/0956797616639301 PMC4899156

[44] Janacsek, K., Fiser, J. & Nemeth, D. The best time to acquire new skills: Age-related differences in implicit sequence learning across the human lifespan. *Dev. Sci.* **15**, 496–505. 10.1111/j.1467-7687.2012.01150.x (2012). 22709399 10.1111/j.1467-7687.2012.01150.x PMC3383816

[45] Greenwald, A. G. & Banaji, M. R. Implicit social cognition: attitudes, self-esteem, and stereotypes. *Psychol. Rev.* **102**, 4–27. 10.1037/0033-295X.102.1.4 (1995). 7878162 10.1037/0033-295x.102.1.4

[46] Diamond, A. Executive functions. *Annu. Rev. Psychol.* **64**, 135–168. 10.1146/ANNUREV-PSYCH-113011-143750 (2013). 23020641 10.1146/annurev-psych-113011-143750 PMC4084861

[47] Miyake, A. et al. The unity and diversity of executive functions and their contributions to complex frontal lobe tasks: A latent variable analysis. *Cogn. Psychol.* **41**, 49–100. 10.1006/cogp.1999.0734 (2000). 10945922 10.1006/cogp.1999.0734

[48] Miyake, A. & Friedman, N. P. The nature and organization of individual differences in executive functions. *Curr. Dir. Psychol. Sci.* **21**, 8–14. 10.1177/0963721411429458 (2012). 22773897 10.1177/0963721411429458 PMC3388901

[49] Otto, A. R., Skatova, A., Madlon-Kay, S. & Daw, N. D. Cognitive control predicts use of Model-based reinforcement learning. *J. Cogn. Neurosci.* **27**, 319–333. 10.1162/JOCN_A_00709 (2015). 25170791 10.1162/jocn_a_00709 PMC4387848

[50] Gray-Burrows, K. et al. A systematic review and meta-analysis of the executive function-health behaviour relationship. *Health Psychol. Behav. Med.* **7**, 253–268. 10.1080/21642850.2019.1637740 (2019). 34040850 10.1080/21642850.2019.1637740 PMC8114370

[51] Allan, J. L., McMinn, D. & Daly, M. A bidirectional relationship between executive function and health behavior: evidence, implications, and future directions. *Front. Neurosci.* **10**, 210626. 10.3389/FNINS.2016.00386/XML/NLM (2016). 10.3389/fnins.2016.00386 PMC4993812 27601977

[52] Allom, V., Mullan, B., Smith, E., Hay, P. & Raman, J. Breaking bad habits by improving executive function in individuals with obesity. *BMC Public. Health*. **18**, 1–8. 10.1186/S12889-018-5392-Y/FIGURES/6 (2018). 10.1186/s12889-018-5392-y PMC5902887 29661241

[53] Badre, D., Kayser, A. S. & D’Esposito, M. Frontal cortex and the discovery of abstract action rules. *Neuron* **66**, 315–326. 10.1016/J.NEURON.2010.03.025/ATTACHMENT/A4E1F36C-24C8-44E4-AD40-68DB03FEE616/MMC1.PDF (2010). 20435006 10.1016/j.neuron.2010.03.025 PMC2990347

[54] Braver, T. S., Reynolds, J. R. & Donaldson, D. I. Neural mechanisms of transient and sustained cognitive control during task switching. *Neuron* **39**, 713–726. 10.1016/S0896-6273(03)00466-5/ASSET/DF9B9870-9322-4DD2-A5CF-C8BFF9B57FD4/MAIN.ASSETS/GR5.JPG (2003). 12925284 10.1016/s0896-6273(03)00466-5

[55] Funahashi, S. & Andreau, J. M. Prefrontal cortex and neural mechanisms of executive function. *J. Physiology-Paris*. **107**, 471–482. 10.1016/J.JPHYSPARIS.2013.05.001 (2013). 10.1016/j.jphysparis.2013.05.001 23684970

[56] Koechlin, E. Prefrontal executive function and adaptive behavior in complex environments. *Curr. Opin. Neurobiol.* **37**, 1–6. 10.1016/J.CONB.2015.11.004 (2016). 26687618 10.1016/j.conb.2015.11.004

[57] Osaka, N. et al. The neural basis of executive function in working memory: an fMRI study based on individual differences. *Neuroimage* **21**, 623–631. 10.1016/J.NEUROIMAGE.2003.09.069 (2004). 14980565 10.1016/j.neuroimage.2003.09.069

[58] Osaka, M. et al. The neural basis of individual differences in working memory capacity: an fMRI study. *Neuroimage* **18**, 789–797. 10.1016/S1053-8119(02)00032-0 (2003). 12667855 10.1016/s1053-8119(02)00032-0

[59] Kondo, H., Osaka, N. & Osaka, M. Cooperation of the anterior cingulate cortex and dorsolateral prefrontal cortex for attention shifting. *Neuroimage* **23**, 670–679. 10.1016/J.NEUROIMAGE.2004.06.014 (2004). 15488417 10.1016/j.neuroimage.2004.06.014

[60] Ambrus, G. G. et al. When less is more: enhanced statistical learning of non-adjacent dependencies after disruption of bilateral DLPFC. *J. Mem. Lang.* **114**, 104144. 10.1016/j.jml.2020.104144 (2020).

[61] Nemeth, D., Janacsek, K., Polner, B. & Kovacs, Z. A. Boosting human learning by hypnosis. *Cereb. Cortex*. **23**, 801–805. 10.1093/cercor/bhs068 (2013). 22459017 10.1093/cercor/bhs068

[62] Borragan, G., Slama, H., Destrebecqz, A. & Peigneux, P. Cognitive fatigue facilitates procedural sequence learning. *Front. Hum. Neurosci.* **10**10.3389/fnhum.2016.00086 (2016). 10.3389/fnhum.2016.00086 PMC4776079 26973501

[63] Smalle, E. H. M., Daikoku, T., Szmalec, A., Duyck, W. & Onen, R. M. Unlocking adults’ implicit statistical learning by cognitive depletion. *Proc. Natl. Acad. Sci. U S A*. **119**, e2026011119. 10.1073/pnas.2026011119 (2022). 34983868 10.1073/pnas.2026011119 PMC8764693

[64] Filoteo, J. V., Lauritzen, S. & Maddox, W. T. Removing the frontal lobes: the effects of engaging executive functions on perceptual category learning. *Psychol. Sci.* **21**, 415–423. 10.1177/0956797610362646 (2010). 20424079 10.1177/0956797610362646 PMC2861791

[65] Park, J., Janacsek, K., Nemeth, D. & Jeon, H-A. Reduced functional connectivity supports statistical learning of temporally distributed regularities. *Neuroimage* **260**, 119459. 10.1016/J.NEUROIMAGE.2022.119459 (2022). 35820582 10.1016/j.neuroimage.2022.119459

[66] Tóth, B. et al. Dynamics of EEG functional connectivity during statistical learning. *Neurobiol. Learn. Mem.* **144**, 216–229. 10.1016/j.nlm.2017.07.015 (2017). 28803867 10.1016/j.nlm.2017.07.015

[67] Virag, M. et al. Competition between frontal lobe functions and implicit sequence learning: evidence from the long-term effects of alcohol. *Exp. Brain Res.* **233**, 2081–2089. 10.1007/s00221-015-4279-8 (2015). 25910995 10.1007/s00221-015-4279-8

[68] Pedraza, F. et al. Evidence for a competitive relationship between executive functions and statistical learning. *Npj Sci. Learn. 2024*. **9**, 1. 10.1038/s41539-024-00243-9 (2024). 10.1038/s41539-024-00243-9 PMC11014972 38609413

[69] Hartley, T. & Burgess, N. Complementary memory systems: competition, Cooperation and compensation. *Trends Neurosci.* **28**, 169–170. 10.1016/j.tins.2005.02.004 (2005). 15808348 10.1016/j.tins.2005.02.004

[70] Smittenaar, P., FitzGerald, T. H. B., Romei, V., Wright, N. D. & Dolan, R. J. Disruption of dorsolateral prefrontal cortex decreases model-based in favor of model-free control in humans. *Neuron* **80**, 914–919. 10.1016/j.neuron.2013.08.009 (2013). 24206669 10.1016/j.neuron.2013.08.009 PMC3893454

[71] Buffington, J., Demos, A. P. & Morgan-Short, K. The reliability and validity of procedural memory assessments used in second Language acquisition research. *Stud. Second Lang. Acquis* **2021**:1–28. 10.1017/S0272263121000127

[72] Farkas, B. C., Krajcsi, A., Janacsek, K. & Nemeth, D. The complexity of measuring reliability in learning tasks: an illustration using the alternating serial reaction time task. *Behav. Res. Methods*. **56**, 301–317. 10.3758/s13428-022-02038-5 (2024). 36604378 10.3758/s13428-022-02038-5 PMC10794483

[73] Siegelman, N., Bogaerts, L. & Frost, R. Measuring individual differences in statistical learning: current pitfalls and possible solutions. *Behav. Res. Methods*. **49**, 418–432. 10.3758/s13428-016-0719-z (2017). 26944577 10.3758/s13428-016-0719-z PMC5011036

[74] Howard, D. V. & Howard, J. H. Age differences in learning serial patterns: direct versus indirect measures. *Psychol. Aging*. **4**, 357–364. 10.1037/0882-7974.4.3.357 (1989). 2803630 10.1037//0882-7974.4.3.357

[75] Janacsek, K. & Nemeth, D. Predicting the future: from implicit learning to consolidation. *Int. J. Psychophysiol.* **83**, 213–221. 10.1016/j.ijpsycho.2011.11.012 (2012). 22154521 10.1016/j.ijpsycho.2011.11.012

[76] Kóbor, A. et al. Statistical learning leads to persistent memory: evidence for one-year consolidation. *Sci. Rep.* **7**, 1–10. 10.1038/s41598-017-00807-3 (2017). 28396586 10.1038/s41598-017-00807-3 PMC5429700

[77] Vékony, T., Ambrus, G. G., Janacsek, K. & Nemeth, D. Cautious or causal? Key implicit sequence learning paradigms should not be overlooked when assessing the role of DLPFC (Commentary on Prutean. *Cortex* **148**, 222–226. 10.1016/j.cortex.2021.10.001 (2022). 34789384 10.1016/j.cortex.2021.10.001

[78] Curtis, C. E. & D’Esposito, M. Persistent activity in the prefrontal cortex during working memory. *Trends Cogn. Sci.* **7**, 415–423. 10.1016/S1364-6613(03)00197-9 (2003). 12963473 10.1016/s1364-6613(03)00197-9

[79] Paneri, S. & Gregoriou, G. G. Top-down control of visual attention by the prefrontal cortex. Functional specialization and long-range interactions. *Front. Neurosci.* **11**, 302586. 10.3389/FNINS.2017.00545/XML/NLM (2017). 10.3389/fnins.2017.00545 PMC5626849 29033784

[80] Phelps, E. A., Hyder, F., Blamire, A. M. & Shulman, R. G. FMRI of the prefrontal cortex during overt verbal fluency. *Https://EprintsNclAcUk* **8**, 561–565. 10.1097/00001756-199701200-00036 (1997). 10.1097/00001756-199701200-00036 9080448

[81] Robinson, G., Shallice, T., Bozzali, M. & Cipolotti, L. The differing roles of the frontal cortex in fluency tests. *Brain* **135**, 2202–2214. 10.1093/BRAIN/AWS142 (2012). 22669082 10.1093/brain/aws142 PMC3381725

[82] Rossi, A. F., Pessoa, L., Desimone, R. & Ungerleider, L. G. The prefrontal cortex and the executive control of attention. *Exp. Brain Res.* **192**, 489–497. 10.1007/S00221-008-1642-Z/METRICS (2009). 19030851 10.1007/s00221-008-1642-z PMC2752881

[83] Fox, C. J., Mueller, S. T., Gray, H. M., Raber, J. & Piper, B. J. Evaluation of a short-form of the Berg card sorting test. *PLoS One*. **8**, e63885. 10.1371/journal.pone.0063885 (2013). 23691107 10.1371/journal.pone.0063885 PMC3653789

[84] Harrison, J. E., Buxton, P., Husain, M. & Wise, R. Short test of semantic and phonological fluency: normal performance, validity and test-retest reliability. *Br. J. Clin. Psychol.* **39**, 181–191. 10.1348/014466500163202 (2000). 10895361 10.1348/014466500163202

[85] Langner, R. et al. Evaluation of the reliability and validity of computerized tests of attention. *PLoS One*. **18**, e0281196. 10.1371/JOURNAL.PONE.0281196 (2023). 36706136 10.1371/journal.pone.0281196 PMC9882756

[86] Piper, B. J. et al. Reliability and validity of neurobehavioral function on the psychology experimental Building Language test battery in young adults. *PeerJ* 2015:e1460. 10.7717/PEERJ.1460/SUPP-4 (2015). 10.7717/peerj.1460 PMC4690381 26713233

[87] Woods, S. P. et al. Action (verb) fluency: Test–retest reliability, normative standards, and construct validity. *J. Int. Neuropsychol. Soc.* **11**, 408–415. 10.1017/S1355617705050460 (2005). 16209421

[88] Fan, J., McCandliss, B. D., Sommer, T., Raz, A. & Posner, M. I. Testing the efficiency and independence of attentional networks. *J. Cogn. Neurosci.* **14**, 340–347. 10.1162/089892902317361886 (2002). 11970796 10.1162/089892902317361886

[89] Berg, E. A. A simple objective technique for measuring flexibility in thinking. *J. Gen. Psychol.* **39**, 15–22. https://doi.org/10.1080/00221309.1948.9918159/ASSET//CMS/ASSET/6C468455-B715-4379-B32B-51E9A0D6DB47/00221309.1948.9918159.FP.PNG (1948). 10.1080/00221309.1948.9918159 18889466

[90] Mueller, S. T. & Piper, B. J. The psychology experiment Building Language (PEBL) and PEBL test battery. *J. Neurosci. Methods*. **222**, 250–259. 10.1016/J.JNEUMETH.2013.10.024 (2014). 24269254 10.1016/j.jneumeth.2013.10.024 PMC3897935

[91] Case, R., Kurland, D. M. & Goldberg, J. Operational efficiency and the growth of short-term memory span. *J. Exp. Child. Psychol.* **33**, 386–404. 10.1016/0022-0965(82)90054-6 (1982).

[92] St-Hilaire, A. et al. Normative data for phonemic and semantic verbal fluency test in the adult French–Quebec population and validation study in alzheimer’s disease and depression. *Clin. Neuropsychol.* **30**, 1126–1150. 10.1080/13854046.2016.1195014 (2016). 27279436 10.1080/13854046.2016.1195014

[93] JASP Team. JASP (Version 0.10) 2019.

[94] Song, S., Howard, J. H. & Howard, D. V. Sleep does not benefit probabilistic motor sequence learning. *J. Neurosci.* **27**(46), 12475–12483 (2007). 18003825 10.1523/JNEUROSCI.2062-07.2007 PMC6673329

[95] Dienes, Z. Using Bayes to get the most out of non-significant results. *Front. Psychol.* **5**, 781. 10.3389/fpsyg.2014.00781 (2014). 25120503 10.3389/fpsyg.2014.00781 PMC4114196

[96] Wagenmakers, E., Wetzels, R., Borsboom, D. & van der Maas, H. L. J. Why psychologists must change the way they analyze their data: the case of psi: comment on bem (2011). *J. Pers. Soc. Psychol.* **100**, 426–432. 10.1037/a0022790 (2011). 21280965 10.1037/a0022790

[97] Preuss, H., Pinnow, M., Schnicker, K. & Legenbauer, T. Improving inhibitory control abilities (ImpulsE)—A promising approach to treat impulsive eating?? *Eur. Eat. Disorders Rev.* **25**, 533–543. 10.1002/ERV.2544 (2017). 10.1002/erv.2544 28901678

[98] Galla, B. M. & Duckworth, A. L. More than resisting temptation: beneficial habits mediate the relationship between self-control and positive life outcomes. *J. Pers. Soc. Psychol.* **109**, 508–525. 10.1037/PSPP0000026 (2015). 25643222 10.1037/pspp0000026 PMC4731333

[99] Costa, K. G., Cabral, D. A., Hohl, R. & Fontes, E. B. Rewiring the addicted brain through a Psychobiological model of physical exercise. *Front. Psychiatry*. **10**, 453840. 10.3389/FPSYT.2019.00600/BIBTEX (2019). 10.3389/fpsyt.2019.00600 PMC6718472 31507468

[100] Allom, V., Mullan, B. & Hagger, M. Does inhibitory control training improve health behaviour? A meta-analysis. *Health Psychol. Rev.* **10**, 168–186. 10.1080/17437199.2015.1051078 (2016). 26058688 10.1080/17437199.2015.1051078

[101] Danner, U. N., Aarts, H., Papies, E. K. & De Vries, N. K. Paving the path for habit change: cognitive shielding of intentions against habit intrusion. *Br. J. Health Psychol.* **16**, 189–200. https://doi.org/10.1348/2044-8287.002005 (2011). 10.1348/2044-8287.002005 21199542

[102] Jahanshahi, M., Obeso, I., Rothwell, J. C. & Obeso, J. A. A fronto-striato-subthalamic-pallidal network for goal-directed and habitual Inhibition. *Nat. Rev. Neurosci.* **16**, 719–732. https://doi.org/10.1038/NRN4038 (2015). 10.1038/nrn4038 26530468

[103] Horvath, K., Nemeth, D., Janacsek, K. & Kóbor, A. Independent and interactive dynamics between statistical learning and inhibitory control 2023. 10.31234/OSF.IO/PTDKR

[104] Vékony, T. et al. Retrieval of a well-established skill is resistant to distraction: evidence from an implicit probabilistic sequence learning task. *PLoS One*. **15**, e0243541. 10.1371/JOURNAL.PONE.0243541 (2020). 33301471 10.1371/journal.pone.0243541 PMC7728172

[105] Fisk, A. D., Derrick, W. L. & Schneider, W. A methodological assessment and evaluation of dual-task paradigms. *Curr. Psychol.* **5**, 315–327. 10.1007/BF02686599 (1986).

[106] Ullman, M. T. The role of declarative and procedural memory in disorders of Language. *Linguist Var.* **13**, 133–154. 10.1075/LV.13.2.01ULL (2013).

[107] Marko, M. et al. Assessment of automatic and controlled retrieval using verbal fluency tasks. *Assessment* **30**, 2198–2211. 10.1177/10731911221117512 (2023). 35979927 10.1177/10731911221117512 PMC10478347

[108] Kavé, G. & Sapir-Yogev, S. Associations between memory and verbal fluency tasks. *J. Commun. Disord*. **83**, 105968. 10.1016/J.JCOMDIS.2019.105968 (2020). 31835071 10.1016/j.jcomdis.2019.105968

[109] Nemeth, D., Janacsek, K. & Fiser, J. Age-dependent and coordinated shift in performance between implicit and explicit skill learning. *Front. Comput. Neurosci.* **7**, 147. 10.3389/fncom.2013.00147 (2013). 24155717 10.3389/fncom.2013.00147 PMC3805033

[110] Gustavson, D. E. et al. Integrating verbal fluency with executive functions: evidence from twin studies in adolescence and middle age. *J. Exp. Psychol. Gen.* **148**, 2104–2119. 10.1037/XGE0000589 (2019). 30896200 10.1037/xge0000589 PMC6754807

[111] Amunts, J., Camilleri, J. A., Eickhoff, S. B., Heim, S. & Weis, S. Executive functions predict verbal fluency scores in healthy participants. *Sci. Rep.* **10**, 1–11. 10.1038/S41598-020-65525-9 (2020). 32636406 10.1038/s41598-020-65525-9 PMC7341845

[112] Troyer, A. K., Moscovitch, M. & Winocur, G. Clustering and switching as two components of verbal fluency: evidence from younger and older healthy adults. *Neuropsychology* **11**, 138–146. 10.1037/0894-4105.11.1.138 (1997). 9055277 10.1037//0894-4105.11.1.138

[113] Freedman, J. L. & Loftus, E. F. Retrieval of words from long-term memory. *J. Verbal Learn. Verbal Behav.* **10**, 107–115. 10.1016/S0022-5371(71)80001-4 (1971).

[114] Shao, Z., Janse, E., Visser, K. & Meyer, A. S. What do verbal fluency tasks measure? Predictors of verbal fluency performance in older adults. *Front. Psychol.* **5**, 89695. 10.3389/FPSYG.2014.00772/BIBTEX (2014). 10.3389/fpsyg.2014.00772 PMC4106453 25101034

[115] Daneman, M. Working memory as a predictor of verbal fluency. *J. Psycholinguist. Res.* **20**, 445–464. https://doi.org/10.1007/BF01067637/METRICS (1991).

[116] Azuma, T. Working memory and perseveration in verbal fluency. *Neuropsychology* **18**, 69–77. 10.1037/0894-4105.18.1.69 (2004). 14744189 10.1037/0894-4105.18.1.69

[117] Conway, A. R. A. et al. Working memory span tasks: A methodological review and user’s guide. *Psychon Bull. Rev.* **12**, 769–786. 10.3758/BF03196772 (2005). 16523997 10.3758/bf03196772

[118] Ecker, U. K. H., Lewandowsky, S. & Oberauer, K. Removal of information from working memory: A specific updating process. *J. Mem. Lang.* **74**, 77–90. 10.1016/J.JML.2013.09.003 (2014).

[119] Friston, K. The free-energy principle: A unified brain theory? *Nat. Rev. Neurosci.* **11**, 127–138. 10.1038/nrn2787 (2010). 20068583 10.1038/nrn2787

[120] Pesthy, O. et al. Intact predictive processing in autistic adults: evidence from statistical learning. *Sci. Rep.* **13**, 1–10. 10.1038/s41598-023-38708-3 (2023). 37481676 10.1038/s41598-023-38708-3 PMC10363128

[121] Crawford, S. L. Correlation and regression. *Circulation* **114**, 2083–2088. 10.1161/CIRCULATIONAHA.105.586495 (2006). 17088476 10.1161/CIRCULATIONAHA.105.586495

[122] Bouton, M. E. A learning theory perspective on lapse, relapse, and the maintenance of behavior change. *Health Psychol.* **19**, 57–63. 10.1037/0278-6133.19.SUPPL1.57 (2000). 10709948 10.1037/0278-6133.19.suppl1.57

[123] Berman, D. E., Dudai, Y., Memory & Extinction Learning Anew, and Learning the New: Dissociations in the Molecular Machinery of Learning in Cortex. Science 2001;291:2417–9. (1979). 10.1126/SCIENCE.1058165 10.1126/science.1058165 11264539

[124] Cohen, G. L. & Sherman, D. K. The psychology of change: Self-affirmation and social psychological intervention. *Annu. Rev. Psychol.* **65**, 333–371. 10.1146/annurev-psych-010213-115137 (2014). 24405362 10.1146/annurev-psych-010213-115137

[125] Tóth-Fáber, E., Janacsek, K., Szőllősi, Á., Kéri, S. & Nemeth, D. Regularity detection under stress: faster extraction of probability-based regularities. *PLoS One*. **16**, e0253123. 10.1371/journal.pone.0253123 (2021). 34129623 10.1371/journal.pone.0253123 PMC8205133

[126] Diekhof, E. K., Geana, A., Ohm, F., Doll, B. B. & Frank, M. J. The straw that broke the camel’s back: natural variations in 17β-Estradiol and COMT-Val158Met genotype interact in the modulation of Model-Free and Model-Based control. *Front. Behav. Neurosci.* **15**, 658769. 10.3389/FNBEH.2021.658769/BIBTEX (2021). 34305543 10.3389/fnbeh.2021.658769 PMC8297616

[127] Joue, G. et al. Sex differences and exogenous Estrogen influence learning and brain responses to prediction errors. *Cereb. Cortex*. **32**, 2022–2036. 10.1093/CERCOR/BHAB334 (2022). 34649284 10.1093/cercor/bhab334

